# Purification and Characterization of a New D-Galactose-Specific Lectin from the Housefly, *Musca domestica*, and Its Antiproliferative Effect on Human K562 and MCF-7 Tumor Cells

**DOI:** 10.1673/031.010.7901

**Published:** 2010-06-29

**Authors:** X Cao, Y Sun, C Wang, B. Zeng

**Affiliations:** Tianjin University of Science and Technology, Tianjin, P.R. China.

**Keywords:** atomic force microscopy, glycoprotein

## Abstract

In the present work, a D-galactose-specific lectin with novel N-terminal sequence was purified from *Musca domestica* L. (Diptera: Muscidae) pupae. The purification was performed using affinity chromatography, ultra-filtration, and HPLC. The haemagglutinating activity of *M. domestica* lectin was specifically inhibited by D-galactose. The haemagglutinating activity of this lectin was stable at temperatures up to 65° C and in pH ranging from 4 to 8. Salts including FeCl_3_ and MnCl_2_ inhibited the haemagglutinating process, whereas NaCl, KCl, CaCl_2_, MgCl_2_, ZnCl_2_, and AlCl_3_ did not. By SDS-PAGE, purified *M. domestica* pupae lectin yielded a single band with a molecular weight of 40 kDa, with or without reduction of β-mercaptoethanol, and it could be stained with Alcian Blue 8 GX. The morphology of purified lectin was observed by atomic force microscopy, which indicated that *M. domestica* lectin was an 8.27 nm high, globular shaped glycoprotein with a 1.41 nm high polysaccharide chain. In addition, antiproliferative activity of this lectin against tumor cells K562 and MCF-7 was determined with a colorimetric assay using 3-(4,5-dimethylthiazol-2-yl)-2,5-diphenyl tetrazolium bromide, which showed that the antiproliferative process was time- and dose-dependent with an IC_50_ of 5.7 and 6.7 at 24 h, 5.5 and 6.4 at 36 h, 5.2 and 6.5 µM at 48 h, respectively.

## Introduction

Lectins are proteins or glycoproteins with the selective ability to bind free or conjugated saccharides in a reversible way via two or more binding sites (Sharon 1993). They recognize sequences of two or more saccharides with specificity toward both inter-residue glycosidic linkages and also anomeric configuration, so that they demonstrate antibacterial and anti-tumor abilities by recognizing residues of glycoconjugates on the surface of cells (Richard et al. 2004; Simone et al. 2006).

Extensive studies have revealed that a number of plants can be used for prevention and/or treatment of cancer. For example, lectins from mushroom species including *Agaricus bisporus, Boletus satanus, Flamulina velutipes*, and *Ganoderma lucidm* have antitumor activities (Wang et al. 1998). Lectins from *Arisaema tortuosum* as well as alga *Dasa villosa* and *Ricinus communis* were shown to have antitumor and/or antiproliferative activities (Wei et al. 1978; Dhuna et al. 2005).

The objectives of the present study are to isolate and purify lectins from the haemalymph of *Musca domestica* (Diptera: Muscidae) pupae and to determine their biochemical properties, morphology, N-terminal sequence, and antiproliferative activity.

## Materials and Methods

### Preparation of crude extract from *M. domestica* lectin

*M. domestica* was supplied by Tianjin Sanitation and Epidemic Prevention Station, Tianjin, P.R. China. Approximately 100 g pupae were ground at 4° C by adding 50 ml buffered insect saline (10 mM Tris/HCl, 130 mM NaCl, 5 mM KCl, pH 7.4) and 1 g phenylthiocarbamide (Sigma, www.sigmaaldrich.com). Haemolymph was extracted for 30 minutes followed by freeze centrifugation (8,000 rpm, 20 min, 4° C). The resulting supernatant was collected for further use.

### Affinity chromatography

To increase the capability of the column and make the affinity chromatography procedure more efficient, 15 ml Sepharose-4B (Pharmacia) was added into 50 ml haemolymph with slow stirring for 1 hour at 4° C. The mixture was then packed into a 1.5 × 22 cm column (Bio-rad, www.bio-rad.com) and washed with 500 ml buffered insect saline with a flow rate of 1 ml/min until no protein was detected in the elute by monitoring the absorbance at 280 nm. Absorptions were then eluted with 0.2 M D-galactose-buffered insect saline. Each fraction was dialyzed extensively against buffered insect saline to remove D-galactose.

### Ultra filtration

The fractions were pooled in an ultra filtration cell (8050, millipore) with a 50 kDa membrane (polyethersulfone, biomax PB, Millipore, www.millipore.com). N2 was used to supply a pressure of 0.1 MPa in the ultra filtration cell in order to keep the flow rate stable at 1 ml/min. The elute group with MW under 50 kDa were collected, dialyzed, and concentrated by ultra filtration with a 3 kDa membrane (Millipore). Collections were freeze-dried before storage.

### Purification by HPLC

Protein collections with MW under 50 kDa were applied to HPLC column (TSK gel Super SW3000, 4.6 mm × 30 cm, TOSOH, www.tosoh.com) at a protein concentration of 1 mg/ml. Buffered insect saline at pH 6.7 was used as the elution buffer at a flow rate of 0.1 ml/min successively, and the absorbance at 280 nm was monitored.

### Assay for properties of lectin Haemagglutinating assay

The Bradford assay was used to determine the total protein concentration of the sample (Bradford 1976). To measure haemagglutinating activity of lectin, 25 µl of a serially diluted sample was mixed with 25 µl 2.5% trypsinized red blood cells of rabbits. A suspension containing 1% (w/v) bovine serum albumin in a well of a V-bottomed micro-titer plate was incubated for 1 h at 37° C. The haemagglutinating titer, defined as the reciprocal of the highest dilution exhibiting haemagglutination, was regarded as one haemagglutinating unit. Specific activity was calculated as the number of haemagglutinating units per mg of protein (Wang et al. 2000).

### Inhibition of haemagglutinating assay

Serial dilutions of carbohydrate samples including lactose, D-galactose, maltose, D-mannose, D-glucose, D-ribose, fructose, N-acetyl-lactosamine, N-acetyl-galactosamine, and N-acetyl-D-glucosamine were prepared in buffered insect saline. All of the dilutions were mixed with an equal volume (25 µl) of a solution of the lectin with 50 haemagglutinating units at room temperature for 30 min, and then 50 µl trypsinized 2.5% rabbit erythrocyte suspension was added into the mixture. The minimum concentration of the sugar in the final reaction mixture, which completely inhibited 50 haemagglutinating units of the lectin preparation, was calculated.

### Effect of temperature and pH on the haemagglutinating activity of *M. domestica* lectin

The heat stability of the haemagglutinating activity of *M. domestica* lectin was determined by incubating lectin solution with 50 haemagglutinating units at different temperatures (15–75° C for 60 min) for 60 min, and the remaining haemagglutinating activity was determined. The effect of pH on *M. domestica* lectin haemagglutinating activity was investigated in an analogous manner at different pH ranging from 3 to 9 for 60 min.

### Effect of metallic ions on haemagglutination

Purified lectin solution with 50 haemagglutination units was incubated for 30 min with 50 mM EDTA. Meanwhile, the lectin sample was dialyzed exhaustively against 0.45 M NaCl, and the haemagglutinating activity was assessed with and without addition of 3–12 mM NaCl, KCl, CaCl_2_, MnCl_2_, MgCl_2_, ZnCl_2_, AlCl_3_, or FeCl_3_.

### SDS-polyacrylamide gel electrophoresis and N-terminal sequence

The molecular weight of lectin was estimated by SDS-PAGE in accordance with the procedure of Laemmli (1970) calibrated using a low molecular mass marker (Shanghai Sangon, www.sangon.com). 20 µg of sample protein were loaded on the SDS-PAGE gels (12%>) and stained with Coomassie brilliant blue R 250.

FAlcian Blue 8 GX was used to stain polysaccharide chain of *M. domestica* lectin according to the method described by David (2002), with modifications. SDS was removed thoroughly by immersing gels in 20% ethanol for 4 hours with continuous shaking. Gels were treated with staining solution (0.5 g Alcian blue dissolved in 2% acetic acid) for 2 hours and destained in ethanol, distilled water and acetic acid (9:10:1).

The N-terminal sequence of the lectin was determined using a Hewlett-Packard HP G1000A Edman degradation unit and an HP 1000 HPLC System (Lam et al. 2001). 10 µl of sample loading solution was added first, and the sample was then loaded below the sample loading solution. The sample tube was rinsed with 20 µl neat TFA that was added to the sample solution. The sample was then loaded onto the HPLC column and was eluted using three solutions: A: aqueous triethylamine/acetonitrile, B: aqueous triethylamine/propanol and C: acetonitrile using a flow rate of 325 µl/min..

### In vitro antiproliferative assay of *M. domestica* lectin using K562 and MCF-7 cells

#### Culture of cells

Human leukemia K562 cells and breast cancer MCF-7 cells (obtained from Tianjin Medical University) were cultivated in RPMI-1640 medium supplemented with 10% fetal bovine serum, 100 U/ml penicillin and 100 µg/ml streptomycin. Human lung fibroblasts (HLF) (obtained from Tianjin Medical University) were ‘grown in Dulbecco's modified eagle's medium, containing 10% fetal bovine serum, 100 U/ml penicillin and 100 µg/ml streptomycin. All the cells were cultivated with 5% CO_2_ at 37° C.

#### Cell viability analysis

Cytotoxic effects on the growth and viability of cells were determined using tetrazolium dye assay, as previously described (Horiuchi et al. 1988). Briefly, K562, MCF-7 and HLF cells at concentration of 3 × 10^4^ cells/ml were plated in 96-microwell plates for 6 h, and then various concentrations (1–11 µM) of *M. domestica* lectin were added. After incubation for 24, 36, and 48 hours, 20 µl of tetrazolium reagent was added to each of the 100 µl culture wells (tetrazolium solution was prepared at 5 mg/ml in PBS, filter sterilized, and stored in the dark at 4° C for a maximum of 1 month). After continued incubation for 3 h at 37° C the water-insoluble formazan dye was solubilized by adding 150 µl DMSO to the culture wells. The plates were then shaken gently for 10 min at room temperature, and the optical density of the wells was determined using an ELISA microplate reader at a test wavelength of 570 nm and a reference wavelength of 690 nm. Control cells were grown under the same conditions without the addition of *M. domestica* lectin. Inhibition (%) was calculated according to the method: (C -T) / C × 100, in which C is the average optical density of the control cells group and T is the average optical density of the *M. domestica* lectin treated group.

### Morphology of *M. domestica* lectin by atomic force microscopy

*M. domestica* lectin was attached via electrostatic interactions by contact with freshly cleaved mica that had been coated with poly L-lysine, a positively charged compound (Chun et al. 2006). After the mica was cleaned with methanol and Milli-Q water, 5 µl of 10^-2^ M poly L-lysine solution was applied and incubated for 30 min. The mica surface was then washed with Milli-Q water and 5 µl *M. domestica* lectin was added. After the sample air-dried, the mica was examined using atomic force microscopy. The experiments in tapping mode of operation were carried out using a scanning probe microscope. Commercial silicon cantilevers nano-probe with a spring constant of 0.03 and 0.1 N/m were used for tapping modes in this study.

## Results

### Purification results

**Figure 1.**
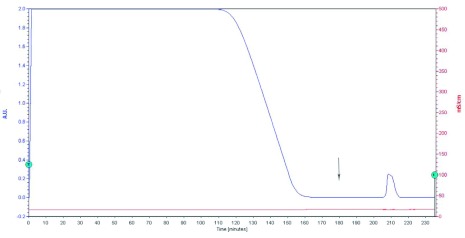
Affinity chromatography of proteins from the haemalymph of *Musca domestica* on a Sepharose-4B column (1.5 × 22 cm). All unbound proteins were eluted from the Sepharose-4B column by using buffered insect saline (pH 7.4) at a flow rate of 1 ml/min until no further proteins were detected at the absorbance of 280 nm after 170 min. The D-galactose specific proteins were obtained by eluting with 0.2 M D-galactose in buffered insect saline (pH 7.4) as the arrow indicated at 180 min. Proteins corresponding to the peak yield at 210 min showed a haemagglutinating activity of 614655 U. High quality figures are available online.

Affinity chromatography using Sepharose-4B was able to absorb lectin effectively from housefly haemolymph. All the proteins without a D-galactose-specific characteristic were eluted out from the column rapidly. Bound fractions were further eluted with 0.2 M D-galactose in buffered insect solution at 180 minutes as shown in Figure 1. A comparatively wide peak at 210 min showed haemagglutinating activity of 614655 U (Table 1).

**Figure 2.**
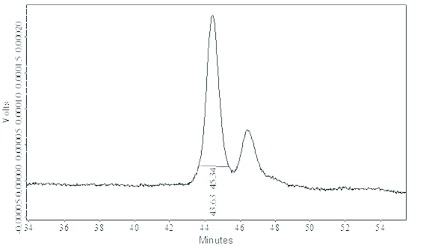
Protein fractions (1 mg/ml) with molecular weight under 50 kDa were applied on a TSK gel HPLC column (Super SW3000 4.6 mm×30 cm). Buffered insect saline pH 6.7 was used as elution with a flow rate of 0.1 ml/min.The first peak, corresponding to the D-galactose specific protein, presented a significant haemagglutinating activity of 483752 U. High quality figures are available online.

After the ultra-filtration process, fractions with a molecular weight under 50 kDa showed a haemagglutinating activity of 521400 U and were further separated by a TSK gel Super SW3000 HPLC column. Two peaks with a molecular weight of 40 kDa and 35 kDa were observed (Figure 2). The 40 kDa peak exhibited a significant haemagglutinating activity of 483752 U (Table 1).

### Properties of *M. domestica* lectin Inhibition of haemagglutinating activity by carbohydrates

A variety of carbohydrates were used to detect the carbohydrate-specific characteristic of *M. domestica* lectin. Total inhibition was observed when D-galactose was used, and the minimum inhibitory concentration was 12.5 mM. No other carbohydrates showed a similar inhibitory effect in this study, which indicated that *M. domestica* lectin was a D-galactose-specific lectin.

### Effects of temperature, pH and metallic ions on haemagglutination

*M. domestica* lectin was stable at temperature up to 65° C, and its haemagglutinating activity was preserved. Meanwhile, the activity was stable at a pH range of 4 to 8. None of the haemagglutinating activities observed showed metallic ion-dependency except Fe^3+^ and Mn^2+^ at concentrations ranging from 3 to 12 mM.

### SDS-PAGE and N-terminal sequence

**Figure 3.**
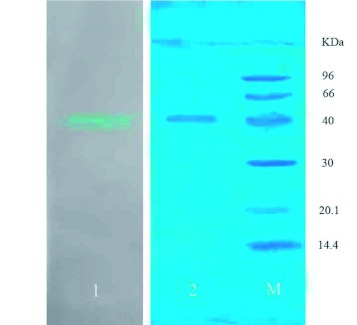
SDS-PAGE. Lane 1: *Musca domestica* lectin stained with Alcian Blue 8 GX. Lane 2: *M. domestica* lectin yielded a single band after staining with Coomassie brilliant blue. The same results were obtained with and without adding β-mercaptoethanol. Lane M: Low molecular mass marker. High quality figures are available online.

After purification on SDS-PAGE gels, *M. domestica* lectin appeared as a single band with and without β- Affinity chromatography of proteins from the haemalymph of *Musca domestica* on a Sepharose-4B column (1.5 × 22 cm). All unbound proteins were eluted from the Sepharose-4B column by using buffered insect saline (pH 7.4) at a flow rate of 1 ml/min until no further proteins were detected at the absorbance of 280 nm after 170 min. The D-galactose specific proteins were obtained by eluting with 0.2 M D-galactose in buffered insect saline (pH 7.4) as the arrow indicated at 180 min. Proteins corresponding to the peak yield at 210 min showed a haemagglutinating activity of 614655 U., which showed that the MW of *M. domestica* lectin was approximately 40 kDa and that there were no subunits in purified *M. domestica* lectin. Lane 1 showed that *M. domestica* lectin could be stained with Alcian Blue 8 GX and indicated that *M. domestica* lectin was a glycoprotein (Figure 3).

**Table 1.**
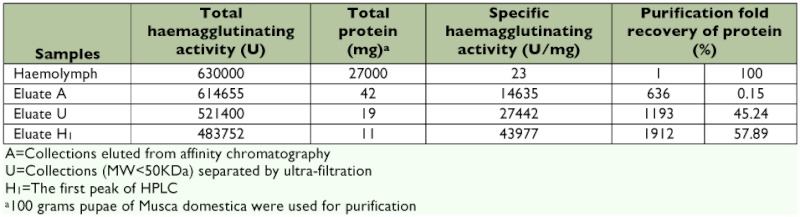
Haemagglutinating assay

The N-terminal sequence of *M. domestica* lectin was not homologous to known lectins isolated from various organisms, but there was slight similarity to some non-lectin proteins (data not shown).

### Antiproliferative effect of *M. domestica* lectin on K562 and MCF-7 cells

**Figure 4.**
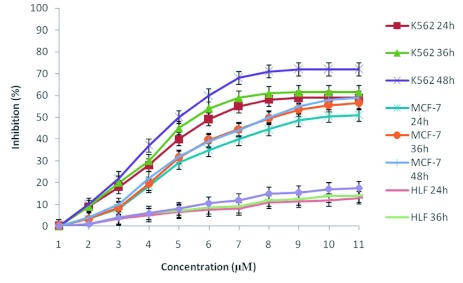
Cell viability was determined by tetrazolium dye assay. K562, MCF-7 and HLF cells at concentration of 3 × 10^4^ cells/ml were plated in 96-microwell plates for 6 h, and then various concentrations (1–11 µM) of *Musca domestica* lectin was added. After incubation for 24, 36, 48 hours respectively, tetrazolium reagent (20 µl) was added to each of the 100 µl culture wells. After continued incubation for 3 h at 37° C, the formed water insoluble formazan dye was solubilized by adding 150 µl DMSO to the culture wells. The plates were then shaken mildly for 10 min at room temperature, and optical density (OD) of the wells were determined using ELISA microplate reader at a test wavelength of 570 nm and a reference wavelength of 690 nm. The viability of cells was expressed as the mean ± SD of three separate experiments (n = 3 for each of the three experiments). HLF were used to examine cytotoxic effect of *M. domestica* lectin on normal cells of human beings. High quality figures are available online.

*M. domestica* lectin inhibited the survival of K562 cells and MCF-7 cells in a time- and dose-dependent manner, while the viability of HLF cells was not significantly affected (Figure 4). The IC_50_ was 5.7 and 6.7 at 24 h, 5.5 and 6.4 at 36 h, 5.2 and 6.5 µM at 48 h, respectively.

### Glycoprotein morphology of *M. domestica* lectin

**Figure 5.**
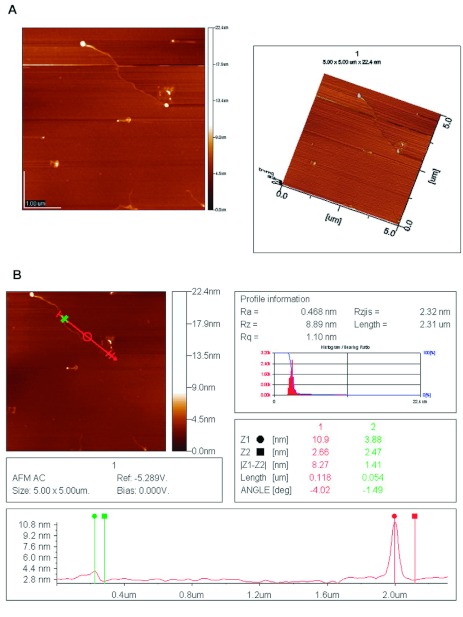
(A) Two- and three-dimensional morphology of *Musca domestica* lectin as revealed by atomic force microscopy. Observation of *M. domestica* lectin morphology showed globular shape of lectins as glycoproteins (bright dots) and tail shaped polysaccharide chains. (B) Measurement of the height of *Musca domestica* lectin. Red line shows the height of the lectin while green shows the height of the polysaccharide chain. It indicates that the heights of lectin and its polysaccharide chain were 8.27 nm and 1.41 nm, respectively. High quality figures are available online.

Two- and three- dimensional structures of *M. domestica* lectin, ascertained by atomic force microscopy, revealed that *M. domestica* lectin was a globular-shaped protein with a tail-shaped polysaccharide chain (Figure 5A). According to the analysis, the height of the glycoprotein was 8.27 nm from peak to valley while the height of the polysaccharide chain was 1.41 nm (Figure 5B).

## Discussion

The lectin isolated from *M. domestica* pupae had an N-terminal amino acid sequence not found in previously reported lectins isolated from many organisms. Its homology to a glutamine transport ATP-binding protein, a putative olfactory receptor, and a protein from *Drosophila melanogaster* was restricted to only small segments of the interior of the molecules. Hence *M. domestica* lectin was a novel lectin.

*M. domestica* lectin was stable at temperatures up to 65° C. This was in sharp contrast to some other lectins. For instance, *Pinus sylvestris* lectin lost all of its hemagglutinating activity at 55° C (Ko et al. 1995) and *Pseudostellaria heterophylla* lectin retained only 50%) of its hemagglutinating activity at 40° C (Peumans et al. 1995). The *M. domestica* lectin, however, was stable with respect to pH variations from 4 to 8 in contrast to some of the lectins that were stable at a wider pH range. *Ganoderma capense* lectin was stable in pH from 3 to 12 (Patrick et al. 2004), and *Agaricus campestris* lectin was stable at pH from 4 to 10 (Sage et al. 1969), for example. *M. domestica* lectin was also distinctive in that its hemagglutinating activity could not be inhibited by a variety of metal chlorides, except MnCl2 and FeCl3.

Similar to some peptides isolated from *Calliphora vicina* (Diptera) (Chernysh et al. 2002), *M. domestica* lectin showed significant antiproliferative activity against K562 and MCF-7 cells, but not against HLF cells in cytotoxic assays. The result concurred with research showing that crude extract from *M. domestica* used as a traditional Chinese treatment exhibited antitumor activity against different tumor cell lines (Hou et al. 2007). How much of the *M. domestica* lectin entering the tumor cells from the incubation medium and the mechanism of action of the lectin against the cells has not been investigated. Since several other lectins can bind to the carbohydrate site on the surface of basophils (Haas et al. 1999) and blood cells (Benjamin et al. 1997), it is likely that the *M. domestica* lectin binds to the cellular membrane of tumor cells and exerts the cytotoxic effect. In future work, it must be clarified whether a peptide or a carbohydrate moiety of the *M. domestica* lectin molecule has biological functions. Lectins have been used for gastrointestinal targeting (Woodle 2000). Some lectins, such as tomato and jacalin lectins, retain their antitumor activity after passage through the gastrointestinal tract (Yu et al. 2001).

Haemagglutinins are considered potentially useful in biochemical and clinical applications, due to their carbohydrate-binding specificities (Chiles et al. 1990; Hori et al. 1996). However, the precise physiological role(s) of these proteins are not yet known. A number of recent reports have suggested that lectin receptors present on the surfaces may function in cell recognition, cell surface adhesion, and symbiosis in invertebrates (Rogers et al. 1993). To provide additional insight, *M. domestica* lectin was examined by Alcian blue staining and by atomic force microscopy. The staining experiment indicated that *M. domestica* lectin was a glycoprotein, and the atomic force microscopy showed that it was a globular-shaped protein with a tail-like polysaccharide chain. Lectins that bind mainly to D-galactose, N-acetyl-galactosamine, and both D-galactose and N-acetyl-galactosamine have been shown to have anticancer activity, and their cytotoxicity differs due to their sugar specificity and glycosylation (Ziska et al. 1991). Although there might be contributions by other components, the present study suggests that the cytotoxic effects of *M. domestica* haemolymph were mediated by this D-galactose-specific *M. domestica* lectin and/or its polysaccharide.

In summary, a novel lectin was purified and characterized from *M. domestica* pupae, appears to be a strong candidate for future applications in lectin research and biomedical applications.

## Abbreviations

HLF: human lung fibroblasts;
K562: leukemia cells;
MCF-7: breast cancer cells
